# *Foni phronimos *- An interview with Edmund D. Pellegrino

**DOI:** 10.1186/1747-5341-5-16

**Published:** 2010-11-09

**Authors:** James Giordano

**Affiliations:** 1 Potomac Institute for Policy Studies, 901 N. Stuart St. Suite 900, Arlington, VA 22203, USA; 2 Oxford Centre for Neuroethics, Uehiro Centre for Practical Philosophy, University of Oxford, OX1, 1PT Oxford, UK

## *Foni phronimos*: (Gr) the voice of one who possesses practical wisdom

One year after the end of his tenure as Chair of the President's Council on Bioethics (PCB), and upon the event of his 90th birthday and 66th anniversary of entering the medical profession, Edmund D. Pellegrino MD, Professor Emeritus of Medicine and Philosophy at Georgetown University, Washington, DC, spoke candidly with *PEHM *Editor-in-Chief James Giordano, about the PCB, a career in medicine and bioethics, and the roles and relationship of the humanities in science and medicine. We invite comments and reflection from the readership - both upon Prof. Pellegrino's thoughts and exposition herein, and upon his work and its impact, writ-large.

• JG. - Professor Pellegrino, I'd like to begin by asking you to describe your work during your tenure as Chair of the President's Council on Bioethics. Specifically, in what ways do you feel that you fostered and cultivated the direction of the council?

• EDP. - I hesitate to speculate on what should have been. I have no criticisms of my predecessors. Nor can I be critical of the new Commission, because their work has just begun, and reflects the needs and tasks placed before its members by the President and his administration. So, I can only speak to what I sought to achieve during my time as Chair. I tried to foster the council as primarily a public education instrument, on the complicated subject of bioethics, which has now become evermore important to public policy, because many of the disputes and differences of opinions are being put into the frame of guidelines, policy and/or law. I truly believe in a democracy. Maybe some people would criticize me for thinking that the general public is more thoughtful than many think they are. I honestly believe that if we set out the alternatives and arguments, people can evaluate them for themselves and a democracy is the only way to allow this to fully occur. So, the first thing on my agenda was to ensure that the function of the Council was not policy formulation as some would have it, but rather education of both the public and the policy makers. In this way, we sought to argue ethically important issues - whatever they happened to be - from a basis of rational consideration of the alternatives that were available, and thus to educate and inform both the government and the people - as the ultimate subjects of ethical decisions.

• JG - Do you feel that you were successful in doing this?

• EDP - That is hard to evaluate at this point, Jim. We do not know the impact of these studies, so I cannot answer that question. That question would be a good starting point from which to look back at the history of previous councils, compare them, and gain insights into how our recommendations - and those of prior councils - affected public and governmental decisions, and use this information to guide and enhance the effectiveness of the present and future councils.

• JG - Do you see the councils as having changed, not only in terms of what you did during your tenure, but throughout its evolution?

• EDP - If you go back to the councils and commissions beginning with the Belmont Report there has been an evolution of sorts. So, for right now, the evolution of the council and its real or potential impact is something that we still cannot describe. It remains a work-in-progress that will reflect its composition and the charge given to it by the administration under which it has been formed.

• JG - Let's talk about that impact for a moment. Personally, when you look back on this, what do you believe is the hallmark achievement of your tenure as Chair of the President's Council?

• EDP -. What I hope will be one of the hallmarks, is as a matter of fact, just what I said - that that the Council was not a podium, but a real forum. We discussed the issues from many sides, examined them, turned them around, and then presented them in clear, brief language. I think that it was equally important that the council produced documents that did not speak down to people, and were relatively free of any professional jargon, at least as much as possible, and I say that with a certain degree of emphasis. If, in retrospect people think that we accomplished at least that much, I believe *that *would be a real achievement.

• JG - You came to the council at a very interesting time, shortly after the turn of the century, subsequent to the Human Genome Project, and Congressionally-declared Decade of the Brain.

• EDP - That is correct.

• JG - Now we are confronting questions arising in nanotechnology, neurotechnology, cybertechnology, as well as many of the older, but still unresolved debates involving genotechnology, stem cells, and cloning. What do you see as the most provocative issues on the horizon, subsequent to your term as Chair of the Council, and now that a new Commission has been formed and becomes active?

• EDP -What do I think is the most pressing and complicated and perhaps the most distressing and publicly significant issue in the field of biomedical ethics? Today, when we speak of an issue in biomedical ethics, we can expect that it will be in the public realm and will very shortly become a policy question. I think the issues that are most fundamental and will continue to be the most decisive are what I call the human life questions. What is the nature of a human being, biologically, philosophically and theologically? Let's face it, there are religious people in the nation - and we must address theological anthropology. What is it to be a human? Is there any distinction between humans and other species? What are those distinctions and how do they affect the ethical decisions that we must make? Are there some things which ought never to be done? I think the decisive response is the question of what it is to be a human being and -at the risk of repeating myself - when you ask these questions, you are asking the kinds of questions that science alone cannot answer. Science can tell us how we work. Science can tell us what we can do to modify those workings. Science can explain the way things are related to each other. We can probe into how they have come about through the use of the experimental method. But, science cannot ever tell us what we *ought *to do, or what we *should *do. This well illustrates the division between the proper realm of science, and the proper realm of philosophy, and by extension philosophical ethics. That is to say, why should we be moral? What are the justifications for being moral? Why do we select one form of morality as opposed to another? How do these realms relate to each other? How do philosophical and theological ethics relate to each other?

• JG - Fifty one years ago, C. P. Snow delivered the *Two Cultures *lectures. There have been those who have said that over the past 50 years both science and humanities have made inroads such that these two cultures are not quite so dissonant, but still, one must admit that science and the humanities certainly occupied distinct places and have had unique paths. In reading your philosophical writings, it has become apparent that your views are very strong with regard to the role of philosophy in medicine, and the philosophy of medicine. I think that is important to discuss, certainly because it does indeed, flavor what and how a moral philosophy is relevant to science and medicine.

• EDP - And I would add, how are they are related to each other in a contemporary world. If you take the view of some, philosophy has no standing. There are philosophers who agree with that, and who would say that philosophy should be the handmaiden of the sciences. That position seems to have taken on a bit of criticism, but nonetheless, I think that it is a major conviction of many people that there is no truth other than scientific truth. I would dare to say that that is metaphysically naive. The idea that there is a real world, and that you can apprehend its truth, by careful thought, has been abandoned by many Post-modern philosophers. Many scientists are equally willing to accept that they cannot grasp whole truth. This is the problem that I have talked about earlier when I referred to philosophical anthropology. Part of this problem arises out of forgetting the medieval lesson of describing the formal object and material object that characterizes an important approach to almost any discipline. Of course, there are those for whom once I mention the word medieval, minds will clap shut, in that they think that any medieval approach is going to be anachronistic. What can I say to that except that maybe I can attempt to reason that the method of science is not the method that you use to examine these philosophical and ethical kinds of questions. That is what Einstein was telling us, in *Out of My Later Life*, a collection of his non-technical essays that so well depicted his respect for the fact that there was another methodology beside that of science. Humans can be looked at from the point of view of physiology, biochemistry, economics, sociology, aesthetics, etc. Each of those disciplines is distinguished from the other by its formal objects, its perspective. Now, you are a neuroscientist and I know you can transcend your neurophysiologic orientation. When you are working at the bench, or in scholarship as a *bona fide *neuroscientist, you are looking at the physical mechanisms of the human brain and nervous system. If you do not do that then you lose scientific credibility and become something else. Yet, you can also reflect philosophically. But you don't have an objective or empirical measurement for those philosophical aspects or questions, and that is the dilemma. So, when one poses philosophical and ethical questions of neuroscience, the answers may, in fact generate questions of their own. Neuroethical answers may remain shrouded in such questions: What do those flashes of light on the fMRI indicate? Activity of cells - yes - but is that thought? We cannot answer that question, at least at the moment. Maybe that will one day be possible. The neurophysiologist has a methodology that distinguishes her from the psychologist, the sociologist, the anthropologist. That is true of all disciplines. Therefore, we can approach ethics as a discipline of its own. The point that I am making is that we need to recover the fact that there are domains that are susceptible to particular methodologies and methodologists. So, we come back to the questions that human beings have asked, "Who am I?" "What am I?" "What is the world about?" We can ask what these questions mean to - and for - a human being who dares to profess to be able to heal by virtue of special knowledge. Medicine cannot resolve these issues, but it must realize their place in the life of the patient.

• JG -Let's take a deeper look at that because there are those who say, particularly now, that medicine is becoming little more than applied science - technology and its applications based upon an imperative for use that swings - and is affected by - the pendulum of progress. This is a contentious domain where ethics and public policy meet. Your views have always emphatically emphasized preserving the humanity of medicine. So, what do you see as the relationship between science, medicine and ethics?

• EDP - Science, that is, the use of the scientific method, tends to confine itself to a study of the physical and perhaps psychological dimensions of human existence. Those aspects that are apprehendable by the method of science. I entered medicine because I wanted to study membrane phenomena, by biophysical means; that was very interesting to me. I did not have any notion that I was going to be finding out what human beings are all about. The realm of ethics is the realm of what is it we *ought *to do and "ought" carries with it the notion of responsibility, accountability and how we reason about a moral question. Quantum mechanics is a sophisticated reflection, but it does not tell you how to live. To do so will take ethics, and ethics is a branch of philosophy because it uses the method of philosophy, i.e. moral contemplation on all aspects of human existence.

It is my strong belief that perhaps now more than ever, there needs to be an intercommunication of the humanities and the sciences. Getting back to medicine, and dealing with the human being, in my view, would therefore need to be distinguished as clinical medicine, that is defined as, and by, the focus upon a human being in the circumstance of illness. What makes medicine the activity it "is", well, that is a metaphysical question. I have argued and written at length on what I think medicine actually is - philosophically and in practice. The point is that medicine is the most scientific of the humanities and the most humane of sciences. It bridges the physical state of the human being with her psychological state, and I daresay with her spiritual state - however we define that to be. That is not just a person's religion, but those transcendent aspects of what she is - and values - beyond the merely material domains of being.

This is not to lessen the importance of the sciences. I think that medicine should be in communication with all the sciences - natural as well as social - as these can add knowledge about what it is to be human. Medicine deals with what it is to "be" in concretely real circumstances of health and illness. So, in the education of the physician, I think we must bring together the science of the human with what it is to be a good human being, and what is the good *for *humans. Toward this end, the humanities are as important as the sciences, but again, this does not - nor should not - degrade science in any way.

In the main, my feeling is that it is important that the physician be well trained in liberal arts. Now, give me a bit of time to go into what I mean by the liberal arts, because it may not be what everybody else means. By the liberal arts I mean those arts that free our minds from the tyranny of other minds. To do this requires critical thinking. A differential diagnosis in medicine is an exercise in dialectics: when I take students to the bedside and ask them to tell me how and why they got to a particular diagnosis, they must go through a process of dialectics. Some time ago, Mortimer Adler wrote a book on dialectics. Important to Adler was the role of the dialectician. One of the examples that Adler used was the formulation of differential diagnosis. As an internist, if I make a contribution to the diagnosis, it is because I am a dialectician: I examine the argument for particular diagnostic claims that are leveraged on both sides and come to the prudential decision for *this *patient. The idea of prudence is classical; as is the idea of dialectics. These ideas have origins in Aristotle's posterior analytics. So, the point is not that science is unimportant to the physician. Equal time should be dedicated to the emphasis on how to think about, evaluate and make prudent decisions because most clinical choices are made without the certainty of having all of the facts, without knowing what the future is going to hold, and having to weigh one thing against another and arrive at what at *this *moment, concretely represents the *right *and *good *decision for a particular patient.

• JG - Two points for further discussion arise from that. Both relate to the fact that you have been called the stalwart of prudence - and virtue ethics - in medicine. If we look at prudence and the idea of the physician-as-*phronimos*, one who possesses and acts with practical wisdom, then we must address those who have denied or refuted *phronesis *in medicine, by claiming that diagnostic medicine is not a *phronetic *activity. This then raises the second point; namely that there are those who have equally castigated virtue as being unrealistic or untenable in a world of progressively pluralist cultures and polyglot values. Where do you see the future of virtue and virtue ethics in contemporary bioethics, and specifically in medicine?

• EDP - Well, first, let me express that I do not feel that I am the guru of virtue ethics. If anyone deserves that title, there are two people, Professor Elizabeth Anscombe in England, and of course Professor Alisdair MacIntyre of Notre Dame. So, I would think of myself as a mere peon in that realm of any such discussion on virtue. But, I do think that virtue ethics will be with us forever, for one simple reason - ethics is the critical evaluation of moral statements and moral decisions. As long as we humans are what we are, it is unlikely that we will produce some post-human state that will transcend accountability and responsibility. In other words, we cannot separate moral actions from the agents that conceive and perform them. Let me give you an example. Let's say, that you are a principlist. I ask you to tell me about beneficence. You say, "Beneficence is to do no harm". I agree, but tell you that this represents the lowest level of beneficence possible; the law requires that. So, then you retort "...you are right, it is the lowest form, but it is doing good for someone even when it causes a certain inconvenience." I say, yes, that is a little bit better, but not by very much. Of course, it transcends the legal because the law does not require anything of you. So, then I turn to somebody else, and she says, "Oh no, beneficence means that you wish well for your fellow human being, even at some cost to yourself." This is a higher level of beneficence. Now, and remember, this is still discussing the same principle, then I talk to Mother Theresa and she says, "These people are wrong; doing good for other people and human beings is the whole purpose of my life and I would do it even with danger to my life, in fact even to the whole giving of my life." Now I must ask - which of these is beneficence? The answer is "all of them"; so the point is that none of these are absolutely right or wrong. It is the character of the human being that allows critical distinction of what a principle means in-effect, and this then takes us back to the question of what it is to be a human being. Surely, there are a number of possible answers to this perdurable question let me offer one: A human being is one who not only can think, but can think responsibly, and therefore can be held accountable. Thus, we cannot get away from character as meaningful to the moral end of human acts. We come back to fact of the virtues, and ultimately to prudence, or to use the terminology of Aristotle - *phronesis*. We cannot get away from prudence, unless we somehow and someday get to the point of being able to predict every aspect of intention and action. Maybe neuroethics, as you've well described it in its "first tradition", will afford science the means to do this at some point in the future, but I doubt it.

• JG -Let's continue in this vein: There are those now who talk about neuroethics, nanoethics, genethics, systems ethics, etc., and you and I have discussed the notions of bioethics, medical ethics and clinical ethics. Do you see these disciplines as distinct? Do you see such distinctions as viable? Or, do you see them as superficial, and in this light, what do you see in terms of the actual portfolios that any or all of these different approaches to ethics would need to share or would need to differentiate?

• EDP - Well, let's begin with the need to share. This is a question of perspectives. Depending upon the kind of question, one may have to go to molecular genetics, neurology, nanoscience, or what have you. Examination of the moral aspects, the nature of the query, by itself, is not served by the methods of those disciplines, per se. Instead, this is served by the methodology of philosophy, and philosophical ethics. The relationship of that method to neuroethics, nanoethics and so on, is such that while the neural, or nano focus leads us to the kinds of problems one may be looking at, one still must ultimately examine these issues ethically and philosophically. So despite these new foci, I think that philosophy, and philosophical ethics are going to be the grounding element that is shared among these fields. It is the paradigm that allows each field to reflect on the realities and abstractions that must be confronted.

• JG - Do you see these sub-disciplines as fracturing the integrity or the focus of philosophical ethics in some way?

• EDP - Perhaps; in spending too much time on the minutiae of the field, there may be a threat or risk of losing the clarity of ethical analysis, and this might muddle some peoples' thinking. But I don't believe that these disciplines can fracture the integrity of particular moral truths. Now, of course, in saying this, I am taking a rather anti-modern stance. I am a moral realist, which is something of heresy today.

• JG - This centers upon the nature of being a moral realist in contemporary times. You have been in medicine for sixty-six years.

• EDP - Correct.

• JG - And have been philosophically active for the majority of those sixty-six years.

• EDP - Also correct.

• JG - Where do you see your particular views as a moral realist situated in the contemporary fabric of society?

• EDP - Moral realism is a thread which still exists. But maybe a more germane question is whether it will - or can - persist. I think that there will always be those that believe that one can apprehend moral reality, and find out about moral truth by study of the real world. So, while moral realism may not necessarily be popular, I think it will persist because it allows for analytic examination that reflects critically upon the experiences of the real world.

• JG - To explore that in further detail: The world is changing in many different ways; some see this as a time for great hope and others view this with trepidation because it represents uncertainty. What do you see as those challenges and tasks, *vis-à-vis*, the ethics of science and medicine?

• EDP - I am going to revert to something that I alluded to earlier. I think the issues and questions are going to be of the most fundamental kind. But, they are not going to be asked in a theoretical way. Instead, they are going to arise from a practical question - shall we or shall we not use a certain biotechnology, for example. Now, like you, I am not against technology. But, the point is that technology does not tell you - to return to the wisdom of Einstein - what you ought to do. I think that the most elemental question is that with each new technology, should we do what we can do, and what are the reasons for doing it or not doing it. Clearly, that is a bioethical conversation. I tend to think that specific questions or issues are going to be centered on this question of what do we do to improve the human being, and human condition, with the biotechnology that we have, and that we might create. I think we need to be mindful of a blind utopian vision. Yes, we can ask ourselves how we might eliminate those things that are impediments to human flourishing, and even happiness. To focus upon generating happiness is far more problematic, at least in my view. Science is the most powerful mutating force in culture today, and will continue to be, unless we destroy ourselves by our own science. Indeed, I think we need to be cautious in how we use our science, lest such terminal consequences may well come about.

• JG - Alas, every utopian coin has a dystopian face.

• EDP - Well said; and now we face the latest utopian urge, which is scientific, and there is nothing wrong with this, but biotechnology cannot substitute for moral and ethical reflection. That is why I believe that Aristotle, Aquinas or Augustine will not - and should not - fade from our view. I've always tried to focus on the perdurable questions, because I think that the proverbial big questions are still the same as they were 3000 years ago. Human nature does not change. I am a conservative in the sense of preserving from the past what is good out of the past and then evaluating it, so as to suggest where we should change. I am not, nor have I ever been a progressivist

• JG - To shift gears a bit; is there anything you wanted to do, but have not, in your career?

• EDP - Yes, there are things that I would have done differently, of course, with the various and different kinds of posts that I have had. If I were to resume those posts today, I would probably spend less time than I have on administration, and spend more time on scholarly efforts because I think that is more long lasting. I also think that the scholarly life is more demanding.

• JG - And in medicine?

• EDP -I would probably avoid the administrative posts and do more scientific research.

• JG - But it was your administrative acumen that enabled Hunterdon Hospital to become a model of what community health care could be and the Health Science Center at Stony Brook University, the model for Research and Science.

• EDP - Correct; I was torn, a love for medicine, the humanities, and bench science. For my personal satisfaction, what would have been more useful? But, you are also right about Hunterdon. When I left that job, I had too much confidence in the durability of the changes we made.

• JG - Some have called the Hunterdon experiment a primordial form of socialized medicine at its finest.

• EDP - That did not distress me at all, if you want to call it a socializing force. I think - and I am sure you know - the word socialize has all kinds of ramifications. If you want to say socialized in the sense of being socially alert, then, yes, but socialized in the sense of the government running it then, no. The two are not the same, as you know.

• JG - You have described yourself as an "academic marine".

• EDP - That is correct.

• JG -Tell me what you mean by that.

• EDP - I did not originally call myself that, my colleagues did, in referring to the fact that I liked to move into a problem where no one has been, and to work the metaphor, take the beach, so to speak, cut through the land mines and prepare it for the people who grow tomatoes. I do not grow tomatoes. I preferred to be the change agent and then hand it over to others to build upon and cultivate.

• JG - So, what's next on the horizon?

• EDP -I am back at the Kennedy Institute of Ethics at Georgetown University, and I want to finish the 2^nd ^Edition of the Philosophy of Medicine, which I envision as a more definitive work. And then I want to do a book on the theory and practice of clinical medicine, which hasn't really been handled well enough in a theoretical fashion. And then maybe a third one on ... a host of personal experiences that I want to call, "*Did Someone Call for a Doctor?" *It would be a series of little vignettes.

• JG - And teaching at the bedside?

• EDP - Oh, yes. I will always do that. I like students and like teaching at the bedside. I think that I will stop teaching only at that time when I cannot deal with the questions anymore. Then I have a moral obligation to stop.

